# Monitor unit calculation for tangential breast treatments: Verification in an anthropomorphic phantom

**DOI:** 10.1120/jacmp.v3i3.2568

**Published:** 2002-06-01

**Authors:** Stephen J. Howlett, Tomas Kron

**Affiliations:** ^1^ Department of Radiation Oncology Newcastle Mater Hospital Locked Bag 7, Hunter Region Mail Centre 2310 NSW Australia

**Keywords:** radiation therapy, dosimetry, anthropomorphic phantom, radiotherapy planning, ionization chamber, monitor unit

## Abstract

This paper presents an anthropomorphic phantom study of dose delivered to a specific point during tangential breast irradiation to verify monitor unit calculations. Measurements were made using a 0.6 cc Farmer type cylindrical ionization chamber in the phantom and compared to calculations made on a three‐dimensional radiotherapy treatment planning system using single digitized contour through to multi slice CT data. A large breast phantom was used for a single field size with a combination of open and wedged fields for three different energies (4, 6, and 18 MV). Solid flat phantom measurements were also performed for comparison. Results showed a lower calculated dose than the dose measured for a fixed number of monitor units where the variations were within a range of 0.8% to 4.5%. Differences were larger for the anthropomorphic phantom than the flat phantom. We conclude that little accuracy is gained from CT based monitor unit calculations compared to those based on digitised contours for this breast treatment but that the dose distributions will be affected. This type of test is recommended as one of a large set, in the commissioning and testing procedures for treatment planning systems.

PACS number(s): 87.52.Df, 87.53.Dq, 87.53.Xd

## INTRODUCTION

Three‐dimensional treatment planning systems (3DRTP) are now common in radiotherapy departments offering improved accuracy and enhanced visualization in the radiotherapy treatment planning process. Breast cancer is one of the most common forms of malignancy occurring in women and radiotherapy has a significant role in the management of this disease. The use of radiotherapy is complicated by the complex geometry and large variability of the target volume for different patients. One expects a more accurate dose calculation from 3DRTP, particularly as the amount of treatment site data input is increased. The absolute dose at the prescription point is of prime importance in radiotherapy treatment planning. Monitor units (MU) are calculated from this point and dose distributions are relative to its value. This study involves the use of an anthropomorphic phantom with breast attachments and an ionization chamber to accurately measure dose at a defined point and compare these measurements to a set of plans derived from a 3D planning system using increasing degrees of complexity from simple single slice contour to full multislice CT data. The use of an ionization chamber allows a higher degree of accuracy which is not achievable with other detector types such as thermoluminescent dosimeters (TLD) or semiconductor diodes. In an earlier study^1^ we reported on phantom treatments involving significant lung heterogeneity. Such measurements are recommended as part of a comprehensive quality assurance (QA) program for treatment planning.^2^ ^,^ ^3^

## MATERIALS AND METHODS

**Figure 1:**
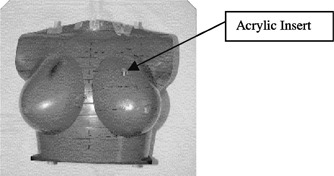
ART anthropomorphic phantom used for measurements.

**Figure 2:**
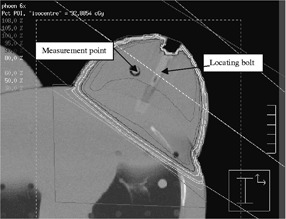
Inferior slice CT plan showing chamber center reference point and dose distribution.

A Farmer type ionization chamber (model 2571, 0.6 cc; 6.3 mm diameter, 24.1 mm length, active volume) traceable to a secondary standard dosimeter was used to measure the dose delivered to an Alderson Radiotherapy (ART) anthropomorphic phantom fitted with two size D breasts, as shown in Fig. 1. The phantom was simulated and planned as per our departmental protocol involving a pair of tangential fields to the left breast, medial to lateral (MD) with gantry angle 306° and lateral to medial (LT) with gantry angle 126°, using a combination of open and wedged fields.^4^ From the simulation procedure a field size of 21.5 cm length×14.0 cm width was used for treatment. The prescription point was chosen in the central slice (0 cm) as halfway between skin and lung on the orthogonal bisector line of the medial to post edge border. Involved lung was included in the plan and heterogeneity accounted for in dose calculations. The central axis was a 0.7 cm distance from the prescription point on this line. No collimator or bed rotations were used so that best reproducibility was achievable. Along the length (superior‐inferior) direction, the left breast was drilled to accommodate the chamber while an acrylic insert was used in its place for CT data acquisition. The point of measurement was located in the slice 4.5 cm inferior to the central axis slice. This point was chosen to give complete coverage in tissue of the chamber and be well in the treatment volume. In our center linear accelerators are calibrated to give 1 MU=1 cGy at depth dmax for reference conditions of 100 cm SSD and 10 cm×10 cm field size at surface, following the IAEA 277 protocol.^5^ In this protocol the actual depth of the chamber measuring point is 5 or 10 cm depending on beam quality. Although different from the AAPM TG 21 protocol,^6^ the resultant calibration values between the two protocols vary only minimally for the photon beams used. Indeed while AAPM TG 51 protocol^7^ displays larger variations,^8^ the measurement analysis reported here will be the same regardless of protocol used, providing the calibration is performed accurately according to the chosen protocol. This is due to the method of comparison described below. The measured doses in the breast were compared to the planned doses derived from a 3D computerized planning system (ADAC Pinnacle 3 v5.0e). The dose calculation method of this system is based on the work of Mackie *et al*.^9^ ^,^ ^10^ The Pinnacle system relies on an input value of dose per monitor unit determined under its recommended reference conditions of 90 cm SSD, 10 cm×10 cm field at isocenter and a depth of 10 cm. All measurements were referenced to this latter setup and the use of the input values obtained at calibration to maintain consistency when comparing to calculated doses. The photon energies, beam quality, and Pinnacle dose per monitor unit values are shown in Table I. These values refer to an output of 1 cGy/MU at $d_{\max}$ as stated above. They were confirmed by solid water flat phantom measurements and Pinnacle calculations. Open fields, 15° and 30° physical wedges (PW), as well as 15° and 30° enhanced dynamic wedges (EDW) were used in irradiations of 100 monitor units per beam. The setup was repeated on different days to assess errors in reproducibility. Measurements were also made using flat solid water phantom material and the same setup parameters to assess the accuracy for normally incident beams. A series of plans were computed based on a single slice digitized contour, five slice digitized contours, and 30 CT slices of 0.5 cm separation (Siemens Somatom CT scanner). These plans incorporated heterogeneity where applicable. Calculations using the water phantom option in the Pinnacle system were used for comparison with the flat phantom solid water measurements. Figure 2 shows the CT plan in 2D with dose distribution and chamber position.

**Table I:** Beam quality and pinnacle reference output values.

Photon energy	Beam quality $\left(d_{20}/d_{10}\right)$	Pinnacle dose/mu (cGy/mu)
4 MV (Varian 600C)	0.572±0.003	0.746±0.002
6 MV (Varian 21EX)	0.576±0.004	0.793±0.002
6 MV (Varian CL1800)	0.581±0.003	0.803±0.002
18 MV (Varian 21EX)	0.666±0.003	0.955±0.002

## RESULTS

Table II shows the results for the 4 MV beam. They are expressed as a ratio of measured over computed where the computed dose was calculated from the full CT data set. Data is given for the central axis point and the –4.5 cm offset point in flat solid water phantom, as well as the point dose for the breast phantom. Table III shows the 6 MV (21EX) results in the same fashion.

**Table II:** Measured doses vs computed doses for flat phantom and Lt breast phantom–4 MV.

4 MV beam	Central axis flat phantom	–4.5 cm offset flat phantom	Lt breast phantom
MD open	1.014±0.003	1.011±0.003	1.019±0.008
MD 15 PW	1.012±0.003	1.020±0.003	1.033±0.009
MD 30 PW	1.016±0.003	1.005±0.003	1.020±0.009
MD 15 EDW	1.014±0.003	1.009±0.003	1.015±0.008
MD 30 EDW	1.013±0.003	1.010±0.003	1.012±0.008
LT open	1.013±0.003	1.011±0.003	1.035±0.009
LT 15 PW	1.013±0.004	1.020±0.004	1.043±0.009
LT 30 PW	1.011±0.004	1.004±0.004	1.031±0.009
LT 15 EDW	1.010±0.004	1.007±0.004	1.031±0.009
LT 30 EDW	1.005±0.004	1.005±0.004	1.029±0.009

**Table III:** Measured doses vs computed doses for flat phantom and Lt breast phantom–6 MV 21EX.

6 MV beam 21EX	Central axis flat phantom	–4.5 cm offset flat phantom	Lt breast phantom
MD open	1.014±0.003	1.012±0.004	1.020±0.008
MD 15 PW	1.007±0.003	1.008±0.003	1.024±0.009
MD 30 PW	1.005±0.003	1.009±0.003	1.037±0.009
MD 15 EDW	1.011±0.004	1.010±0.004	1.024±0.009
MD 30 EDW	1.009±0.003	1.009±0.004	1.025±0.009
LT open	1.015±0.004	1.013±0.004	1.022±0.009
LT 15 PW	1.002±0.003	0.999±0.003	1.033±0.009
LT 30 PW	0.998±0.003	1.004±0.003	1.040±0.009
LT 15 EDW	1.012±0.004	1.010±0.004	1.022±0.009
LT 30 EDW	1.009±0.004	1.009±0.004	1.024±0.009

Table IV shows the other 6 MV results from the CL1800 machine, while Table V displays the 18MV(21EX) comparisons.

**Table IV:** Measured doses vs computed doses for flat phantom and Lt breast phantom–6 MV CL1800.

6 MV beam CL1800	Central axis flat phantom	–4.5 cm offset flat phantom	Lt breast phantom
MD open	1.004±0.003	1.002±0.003	1.015±0.008
MD 15 PW	1.008±0.004	1.017±0.004	1.022±0.009
MD 30 PW	1.002±0.003	1.014±0.004	1.020±0.008
LT open	1.001±0.003	1.001±0.003	1.035±0.009
LT 15 PW	1.008±0.004	1.016±0.004	1.045±0.009
LT 30 PW	0.998±0.003	1.010±0.004	1.040±0.009

**Table V:** Measured doses vs computed doses for flat phantom and Lt breast phantom–18 MV 21EX.

18 MV beam 21EX	Central axis flat phantom	–4.5 cm offset flat phantom	Lt breast phantom
MD open	1.008±0.004	1.015±0.004	1.020±0.008
MD 15 PW	1.002±0.003	1.012±0.004	1.008±0.008
MD 30 PW	1.005±0.003	1.010±0.003	1.011±0.008
MD 15 EDW	1.008±0.004	1.010±0.004	1.018±0.008
MD 30 EDW	1.008±0.004	1.016±0.004	1.021±0.009
LT open	1.009±0.004	1.019±0.004	1.019±0.008
LT 15 PW	1.005±0.003	1.014±0.004	1.018±0.008
LT 30 PW	1.001±0.003	1.012±0.004	1.022±0.009
LT 15 EDW	1.008±0.004	1.015±0.004	1.019±0.008
LT 30 EDW	1.008±0.004	1.019±0.004	1.022±0.009

Calculations were also made for central axis dose using single digitized contour and five digitized contours. These were compared to the 30 CT slice calculations and the resulting calculated dose for 100 MU delivered are shown in Table VI for three energies with open and 15° wedges.

**Table VI:** Computed doses on central axis for Lt breast phantom–4, 6, and 18 MV.

Energy	Beam	30 CT slice	5 contour	1 contour
4 MV	MD open	82.1	81.0	82.2
	MD 15 PW	62.1	61.3	62.2
	LT open	92.7	92.2	92.7
	LT 15 PW	69.9	69.6	69.9
6 MV	MD open	86.7	85.8	86.7
(21EX)	MD 15 PW	62.4	61.8	62.5
	LT open	96.2	95.9	96.0
	LT 15 PW	69.5	69.4	69.4
18 MV	MD open	101.2	100.6	101.3
	MD 15 PW	79.1	78.7	79.2
	LT open	106.9	106.8	107.0
	LT 15 PW	83.7	83.7	83.8

## DISCUSSION

The results overall give confidence in the planning system monitor unit calculations for breast treatments. The central axis and –4.5 cm offset flat phantom measurements are within our accepted range of ±2%3 and most are closer to ±1%. When the same setup parameters are used on the breast phantom the accuracy is not as good. We attribute this to the breast shape which necessitates non‐normally incident beams, to setup variability, and to a lesser extent the heterogeneity effects from the lung volume involved. All breast phantom measurements are <5% of calculated dose, with most being <3%. There is a tendency for the LT beam variations to be larger than the MD beam but this is not significant. This could be explained by the fact that the SSD is further from 90 cm for the LT (95.5 cm) than the MD (92.5 cm). At 90 cm the Pinnacle model parameters would be most accurate and hence give more accurate mu calculations.

With only one exception, the results exceed 1.000 which indicate that the planning system overestimates mu or underestimates dose to the points of measurement. Starkschall *et al*.^11^ reported a similar finding and attributed this to different ray tracing methods between the beam commissioning mode and the treatment planning mode of the Pinnacle system. They increased the machine output value (dose per mu) by 0.5% to remove this systematic effect. This correction would not apply to the flat phantom results presented here as they were calculated using the water phantom option and no differences were found between the physics and planning modes. However, the left breast phantom results are from CT data sets and would be improved by such a correction. The difficulty in our center relates to digitized input data and contour defined CT data where this change would no longer apply and therefore the correction would not be required. Hence, our center has not made this correction.

The computed doses in Table VI show that regardless of breast shape input method acceptable monitor unit calculations are obtained for treatment purposes. Indeed our department still plans breast treatment by digitized contour outlines and occasionally uses CT acquired data. However, the latter offers more accurate 3D dose information, which is important for accurate lung and heart dose as well as techniques such as dynamic mlc.

## CONCLUSION

This work has assessed the accuracy of MU calculations by the Pinnacle planning system for a particular breast treatment by using an anthropomorphic breast phantom and an ionization chamber. Four different beam energies from three different machines were tested with open and wedged fields. Overall the measurements confirmed the dose calculations within an accuracy of <5% with most measurements <3%. This type of test is an important part of the chain of quality assurance procedures to accurately validate a treatment planning system.^2^ ^,^ ^3^ For the prescription dose an accurately obtained single contour would give quite acceptable accuracy for the dose to prescription point when compared to multicontour digitized plans or multisliced CT plans for this treatment setup. The dose distribution, however, would be unreliable off central axis and users should be cautioned to use volume expansion on single contour input only for dose information contained within the single contour and not for any points out of this area. Certainly dose prescription points should only be chosen from a contour which has been obtained from the patient, otherwise monitor unit calculations will be inaccurate. Further work is intended to investigate other treatment sites.

## References

[1] S. Howlett , T. Kron , N. Xuan Ku , and C. Hamilton , “Monitor unit calculations using a 3D computerised treatment planning system: verification in an anthropomorphic phantom” Australas. Phys. Eng. Sci. Med. 22, 163–165 (1999). 10740889

[2] B. Fraass , K. Doppke , M. Hunt , G. Kutcher , G. Starkschall , R. Stern , and J. Van Dyk , “American Association of Physicists in Medicine Radiation therapy Committee Task Group 53: Quality assurance for clinical radiotherapy treatment planning,” Med. Phys. 25, 1773–1829 (1998). 9800687 10.1118/1.598373

[3] J. Van Dyk , R. B. Barnett , J. E. Cygler , and P. C. Shragge , “Commissioning and quality assurance of treatment planning computers,” Int. J. Radiat. Oncol., Biol., Phys. 26, 261–273 (1993). 8491684 10.1016/0360-3016(93)90206-b

[4] P. Cross , D. J. Joseph , J. Cant , S. G. Copper , and J. W. Denham , “Tangential Breast Irradiation: Simple Improvements” Int. J. Radiat. Oncol., Biol., Phys. 23, 433–442 (1992). 1587767 10.1016/0360-3016(92)90765-a

[5] IAEA (International Atomic Energy Agency) , Absorbed Dose Determination in Photon and Electron Beams. An International code of Practice, Technical Report Series No. 277 (IAEA, Vienna, 1987).

[6] R. J. Schulz , P. R. Almond , J. R. Cunningham , J. G. Holt , R. Loevinger , K. A. Wright , R. Nath , and G. D. Lempert , “American Association of Physicists in Medicine Radiation Therapy Committee Task Group 21: A protocol for the determination of absorbed dose from high energy photon and electron beams,” Med. Phys. 10, 741–771 (1983). 6419029 10.1118/1.595446

[7] P. R. Almond , P. J. Biggs , W. F. Hanson , M. Saiful Huq , R. Nath , and D. W. O. Rogers , “AAPM's TG‐51 protocol for clinical reference dosimetry of high‐energy photon and electron beams,” Med. Phys. 26, 1847–1870 (1999). 10505874 10.1118/1.598691

[8] M. Saiful Huq and P. Andreo , “Reference dosimetry in clinical high‐energy photon beams: comparison of the AAPM TG‐51 and AAPM TG‐21 dosimetry protocols,” Med. Phys. 28, 46–54 (2001). 11213922 10.1118/1.1333745

[9] T R. Mackie , J. W. Scrimger , and J. J. Battista , “A convolution method of calculating dose for 15MV *x*‐rays,” Med. Phys. 12, 188–196 (1985). 4000075 10.1118/1.595774

[10] N. Papanikolaou , T. R. Mackie , C. Meger‐Wells , M. Gehring , and P. Reckwerdt , “Investigation of the convolution method for polyenergetic spectra,” Med. Phys. 20, 1327–1336 (1993). 8289713 10.1118/1.597154

[11] G. Starkschall , R. Steadham , N. Wells , L. O'Neill , L. Miller , and I. Rosen , “On the need for monitor unit calculations as part of a beam commissioning methodology for a radiation treatment planning system,” J. Appl. Clin. Med. Phys. 1, 86–94 (2000). 11674822 10.1120/jacmp.v1i3.2640 PMC5726167

